# Pyrogallol protects against influenza A virus‐triggered lethal lung injury by activating the Nrf2–PPAR‐γ–HO‐1 signaling axis

**DOI:** 10.1002/mco2.531

**Published:** 2024-04-12

**Authors:** Beixian Zhou, Linxin Wang, Sushan Yang, Yueyun Liang, Yuehan Zhang, Xuanyu Liu, Xiping Pan, Jing Li

**Affiliations:** ^1^ The People's Hospital of Gaozhou Gaozhou China; ^2^ Cancer Center, Integrated Hospital of Traditional Chinese Medicine Southern Medical University Guangzhou China; ^3^ Guangzhou Laboratory Guangzhou China; ^4^ State Key Laboratory of Respiratory Disease National Clinical Research Center of Respiratory Disease Guangzhou Institute of Respiratory Health Institute of Chinese Integrative Medicine Guangdong‐Hongkong‐Macao Joint Laboratory of Infectious Respiratory Disease the First Affiliated Hospital of Guangzhou Medical University Guangzhou Medical University Guangzhou China

**Keywords:** acute lung injury, heme oxygenase 1 (HO‐1), influenza A virus, pyrogallol, retinoic acid inducible gene I (RIG‐I), type I interferons

## Abstract

Pyrogallol, a natural polyphenol compound (1,2,3‐trihydroxybenzene), has shown efficacy in the therapeutic treatment of disorders associated with inflammation. Nevertheless, the mechanisms underlying the protective properties of pyrogallol against influenza A virus infection are not yet established. We established in this study that pyrogallol effectively alleviated H1N1 influenza A virus‐induced lung injury and reduced mortality. Treatment with pyrogallol was found to promote the expression and nuclear translocation of nuclear factor erythroid‐2‐related factor 2 (Nrf2) and peroxisome proliferator‐activated receptor gamma (PPAR‐γ). Notably, the activation of Nrf2 by pyrogallol was involved in elevating the expression of PPAR‐γ, both of which act synergistically to enhance heme oxygenase‐1 (HO‐1) synthesis. Blocking HO‐1 by zinc protoporphyrin (ZnPP) reduced the suppressive impact of pyrogallol on H1N1 virus‐mediated aberrant retinoic acid‐inducible gene‐I‐nuclear factor kappa B (RIG‐I–NF‐κB) signaling, which thus abolished the dampening effects of pyrogallol on excessive proinflammatory mediators and cell death (including apoptosis, necrosis, and ferroptosis). Furthermore, the HO‐1‐independent inactivation of janus kinase 1/signal transducers and activators of transcription (JAK1/STATs) and the HO‐1‐dependent RIG‐I‐augmented STAT1/2 activation were both abrogated by pyrogallol, resulting in suppression of the enhanced transcriptional activity of interferon‐stimulated gene factor 3 (ISGF3) complexes, thus prominently inhibiting the amplification of the H1N1 virus‐induced proinflammatory reaction and apoptosis in interferon‐beta (IFN‐β)‐sensitized cells. The study provides evidence that pyrogallol alleviates excessive proinflammatory responses and abnormal cell death via HO‐1 induction, suggesting it could be a potential agent for treating influenza.

## Graphical abstract

Schematic diagram depicting the underlying mechanism by which pyrogallol protected against H1N1 virus‐mediated excessive inflammation and lethal lung injury.

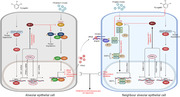

## 1 INTRODUCTION

Influenza A virus infection, which affects the respiratory system is the most common acute lung disease, causing symptoms ranging from mild to severe.^1^ Frequently, influenza‐related deaths are the result of rapid progression from acute lung injury (ALI) to acute respiratory distress syndrome (ARDS).^2^ An increasing number of studies revealed that the combination of antiviral agents with immunomodulators could reduce the mortality induced by pandemic influenza strains or even highly pathogenic influenza subtypes (e.g., H5N1),^3^, ^4^ which highlights the critical role of both viral factors and overwhelming inflammation in the development of influenza‐associated severe ALI. However, few medications are available for the preventive or therapeutic treatment of individuals with influenza‐associated excessive inflammation. Thus, the development of novel agents to treat influenza virus‐triggered intense immune reactions and thereby avoid life‐threatening ALI progression is becoming increasingly important.

Naturally occurring compounds in fruits and herbs provide a diverse array of pharmacological options for the prophylactic or therapeutic treatment of influenza infections that lead to severe conditions. Plant polyphenols are highly abundant compounds that exhibit significant potential antitumor, antioxidant, antiviral, and anti‐inflammatory activities.^5^ Vegetable‐ and fruit‐derived pyrogallol, a polyphenol compound with three hydroxy groups attached to positions 1, 2, and 3 of a benzene ring, shows multiple pharmacological properties against a range of diseases, such as anticancer, anti‐inflammatory, and antibacterial activities.^6^, ^7^, ^8^ It has been reported that pyrogallol exerted these biological activities because it could act on a variety of molecular targets, including Nrf2, p38 MAPK, p53, and p‐AKT.^9^, ^10^, ^11^ In addition, pyrogallol can serve as a superoxide anion generator and also a glutathione depletor, resulting in growth inhibition or apoptosis in several cancer cells.^12^, ^13^ Moreover, pyrogallol and compounds with a pyrogallol moiety possess superoxide radical scavenging activity that could have health‐promoting benefits.^14^ Pyrogallol has the potential to be an electrophile warhead that contributes to covalent inhibitors against the SARS‐CoV‐2 3CL protease.^15^ Interestingly, our previous study showed that pyrogallol treatment promoted the therapeutic effects of human umbilical cord mesenchymal stem cells against lung damage and inflammation caused by LPS.^10^ Nevertheless, the protective properties of pyrogallol against influenza A virus‐induced immune reaction disorders and ALI, along with the corresponding mechanisms, remain unclear.

RIG‐I is a member of RLR family that can recognize influenza virus‐derived 5′ tri‐phosphate RNA with its C‐terminal domain.^16^ Upon stimulation with viral RNA, the interaction between N‐terminal caspase activation and recruitment of RIG‐I and mitochondrial antiviral signaling protein results in IRF3, IRF7, and NF‐κB activation, which assists in raising the generation of proinflammatory cytokines and type I IFNs to induce antiviral immunity.^16^ RIG‐I is a key player in defending against viruses, include influenza A virus.^17^ RIG‐I deficiency led to impaired viral clearance and lower protection against a lethal influenza virus challenge.^18^ In addition to its critical role in antiviral action, activation of RIG‐I concomitantly boosted proinflammatory cytokines and chemokines, including IL‐6, TNF‐α, and CXCL10.^19^ Of note, hyperinduction of proinflammatory mediators (also known as “cytokine storms”) could lead to a detrimental outcome.^20^ The downstream proinflammatory cytokines of RIG‐I signaling were found to be substantially increased in influenza virus‐infected patients with ARDS.^19^, ^20^ Some research, however, revealed that RIG‐I is not required for protection against lethal doses of influenza virus and that the redundant RIG‐I signaling could be a factor in increasing the severity of influenza‐associated lung damage.^21^, ^22^ The discrepancy between these reports may be attributed to the differences in the extent of the inflammatory reaction elicited by different viral strains or different doses of virus inoculation. Therefore, novel strategies are necessary to minimize the inflammation extent mediated by RIG‐I signaling as well as alleviate excessive inflammation‐associated ALI.

Type I IFNs (IFN‐α/β) are the main products of RIG‐I signaling, the signal of which is transmitted by interaction with the receptor chains IFNR1 and IFNR2 to activate the JAK–STAT signaling cascades.^23^ Upon activation, the downstream proteins STAT1, STAT2, and IRF9 form a heterotrimer (termed “ISGF3”) that is translocated to the nucleus for binding to the IFN‐stimulated response element (ISRE), contributing to the initiation of expression of a huge number of genes, which exert antiviral effects and restrict viral replication.^23^ The anti‐influenza virus mechanisms by which type I IFNs exert antiviral effects include inhibition of viral entry by IFITM3,^24^ degradation of viral RNA by OAS1, and limitation of progeny virus release by viperin.^25^ However, type I IFN signaling is linked to excessive proinflammatory reactions and exacerbates apoptosis, resulting in influenza virus‐mediated ALI and high morbidity.^26^ Similarly, in H1N1 virus‐infected patients who experienced ARDS, ARDS was induced by the induction of TRAIL in an IFN‐β‐dependent manner.^27^ Moreover, our earlier investigation showed that IFN‐β pretreatment amplified influenza virus‐induced proinflammatory responses, resulting in severe pneumonia and ALI.^28^, ^29^ Based on these observations, the immunomodulatory effects of IFNs could contribute to a detrimental outcome in influenza diseases. Therefore, the development of novel agents to mitigate the disease‐promoting effects of IFNs is of great importance.

However, the impact of pyrogallol on RIG‐I signaling and the detrimental effects caused by type I IFN during influenza A virus infection, as well as the underlying mechanism, remain unclear. In the present study, we aimed to examine the effects of pyrogallol on viral replication and proinflammatory reactions triggered by the influenza virus and to reveal the underlying mechanisms.

## 2 RESULTS

### 2.1 Pyrogallol alleviates H1N1 virus‐induced ALI

**FIGURE 1:**
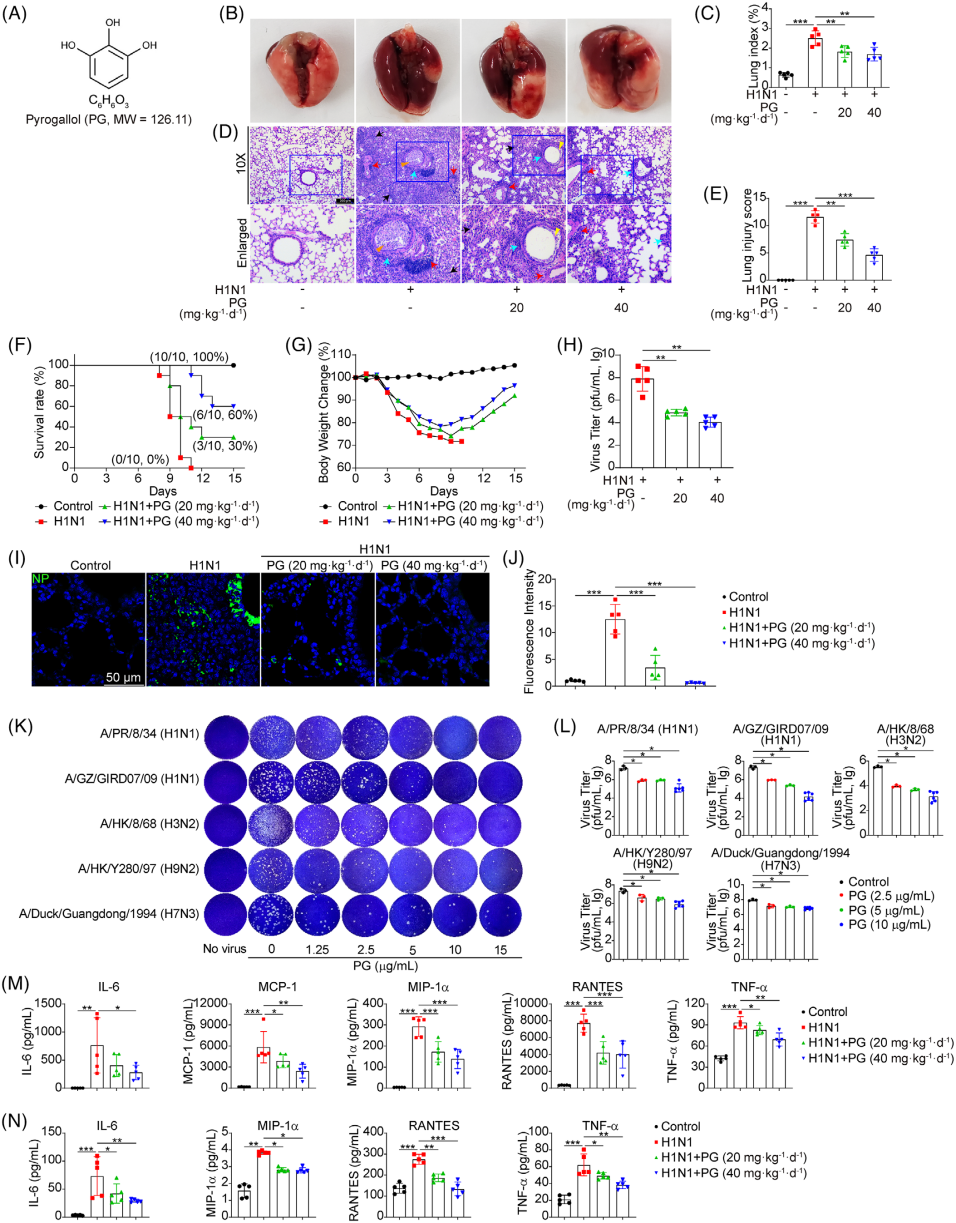
Pyrogallol protects against H1N1 virus‐induced ALI in vivo. (A) Chemical structure of pyrogallol. (B) Gross anatomy of H1N1 virus‐infected lungs. (C) Lung index (lung/body weight ratio). (D) H&E staining of lung tissues (×100, scale bar = 200 µm). Black arrows: alveolitis; red arrows: lung vasculitis; blue arrows: inflammation around the bronchioles; orange triangle: bronchiolitis; yellow triangle: bronchiolar epithelial sloughing. (E) The lung injury was scored. (F and G) Kaplan–Meier survival curve (F) and changes in body weight (G) in mouse‐adapted A/FM/1/47(H1N1)‐infected mice (*n* = 10 mice per group). (H) Viral titers in the lung tissues. (I) Immunofluorescence staining was used to measure NP expression (green). (J) NP's relative fluorescence intensities. (K) Plaque reduction assay of pyrogallol against a series of influenza virus strains. (L) Released progeny viruses in the supernatant. (M and N) Proinflammatory cytokines in the lung tissue homogenates (M) and serum (N). ^*^ *p* < 0.05, ^**^ *p* < 0.01, ^***^ *p* < 0.001.

Our initial objective was to investigate whether pyrogallol might help alleviate ALI induced by the H1N1 virus in vivo. Anatomic pathology examination revealed that mice infected with the H1N1 virus experienced widespread pulmonary edema and bleeding at day 7 postinfection (p.i.), whereas pyrogallol treatment clearly reduced these pathological morphologies (Figure 1B). Concomitantly, the lung index reflecting lung inflammation and injury was also conformably decreased by pyrogallol treatment (Figure 1C). Hematoxylin and eosin (H&E) staining revealed that H1N1 virus infection induced severe pulmonary histopathological changes, including bronchiolitis, vasculitis, alveolar destruction, and diffuse lung parenchymal inflammation, all of which was significantly alleviated by pyrogallol administration (Figure 1D). Also, the high lung pathological score in the group with H1N1 virus infection was also decreased by pyrogallol administration (Figure 1E). Moreover, survival analysis showed that all H1N1 virus‐infected mice died (zero out of 10), whereas the survival rates with pyrogallol treatment (20 and 40 mg·kg^−1^·day^−1^) were increased to 30% (three out of 10) and 60% (six out of 10), respectively (Figure 1F). The mouse body weight following H1N1 virus infection steadily reduced to a minimum at day 10, while the body weight in the pyrogallol treatment groups (20 and 40 mg·kg^−1^·day^−1^) increased gradually (Figure 1G). We postulated that the protective properties of pyrogallol against ALI induced by H1N1 virus were attributed to its antiviral properties. As expected, we observed that pyrogallol reduced virus titers (Figure 1H) and the viral antigen NP expression (Figures 1I and J) in the lungs.

Moreover, we found that pyrogallol treatment effectively suppressed cytopathic effects (CPEs) induced by five strains of influenza, namely A/PR/8/34 (H1N1), A/GZ/GIRD07/09 (H1N1), A/HK/8/68 (H3N2), A/HK/Y280/97 (H9N2), and A/Duck/Guangdong/1994 (H7N3), with IC_50_ and SI values in the range of 0.70 ± 0.06–1.48 ± 0.06 and 5.93 ± 0.26–14.00 ± 6.64 µg/mL, respectively (Table 1). Simultaneously, the plaque reduction assay and the progeny virus production assay showed that pyrogallol significantly decreased viral plaque formation (Figure 1K) and progeny virus production (Figure 1L). In addition to the virus itself, excessive inflammation is also believed to contribute to the severity of influenza disease.^20^ We found that pyrogallol treatment considerably reduced the enhanced expression of proinflammatory cytokines and chemokines induced by the H1N1 virus in the lung homogenates (Figure 1M) and serum (Figure 1N). Thus, the protective properties of pyrogallol against ALI induced by the H1N1 virus may be attributed to its antiviral properties and anti‐inflammatory effects.

**TABLE 1:** Antiviral activity of pyrogallol against influenza viruses.

	Pyrogallol (µg/mL)			Oseltamivir (µg/mL)		
Virus strain	TC_50_ ^a^	IC_50_ ^b^	SI^c^	TC_50_	IC_50_	SI
A/PR/8/34(H1N1)	8.95 ± 0.36	0.74 ± 0.24	14.00 ± 6.64	>1000	1.47 ± 0.23	>1000
A/GZ/GIRD07/09(H1N1)	8.95 ± 0.36	0.75 ± 0.08	12.14 ± 1.90	>1000	1.68 ± 0.11	>1000
A/HK/8/68(H3N2)	8.95 ± 0.36	1.48 ± 0.06	5.93 ± 0.26	>1000	8.21 ± 0.51	>200
A/HK/Y280/97(H9N2)	8.95 ± 0.36	0.70 ± 0.06	12.77 ± 0.70	>1000	9.83 ± 0.33	>200
A/Duck/Guangdong/1994 (H7N3)	8.95 ± 0.36	1.44 ± 0.75	10.62 ± 3.40	>1000	9.33 ± 1.59	>100

^a^ TC_50_ was determined using an MTT assay.

^b^ IC_50_ was determined by cytopathic effect reduction.

^c^ The selectivity index (SI) was determined as the TC_50_/IC_50_ ratio.

### 2.2 Pyrogallol activates the Nrf2–PPAR‐γ–HO‐1 signal axis in H1N1 virus‐infected cells

**FIGURE 2:**
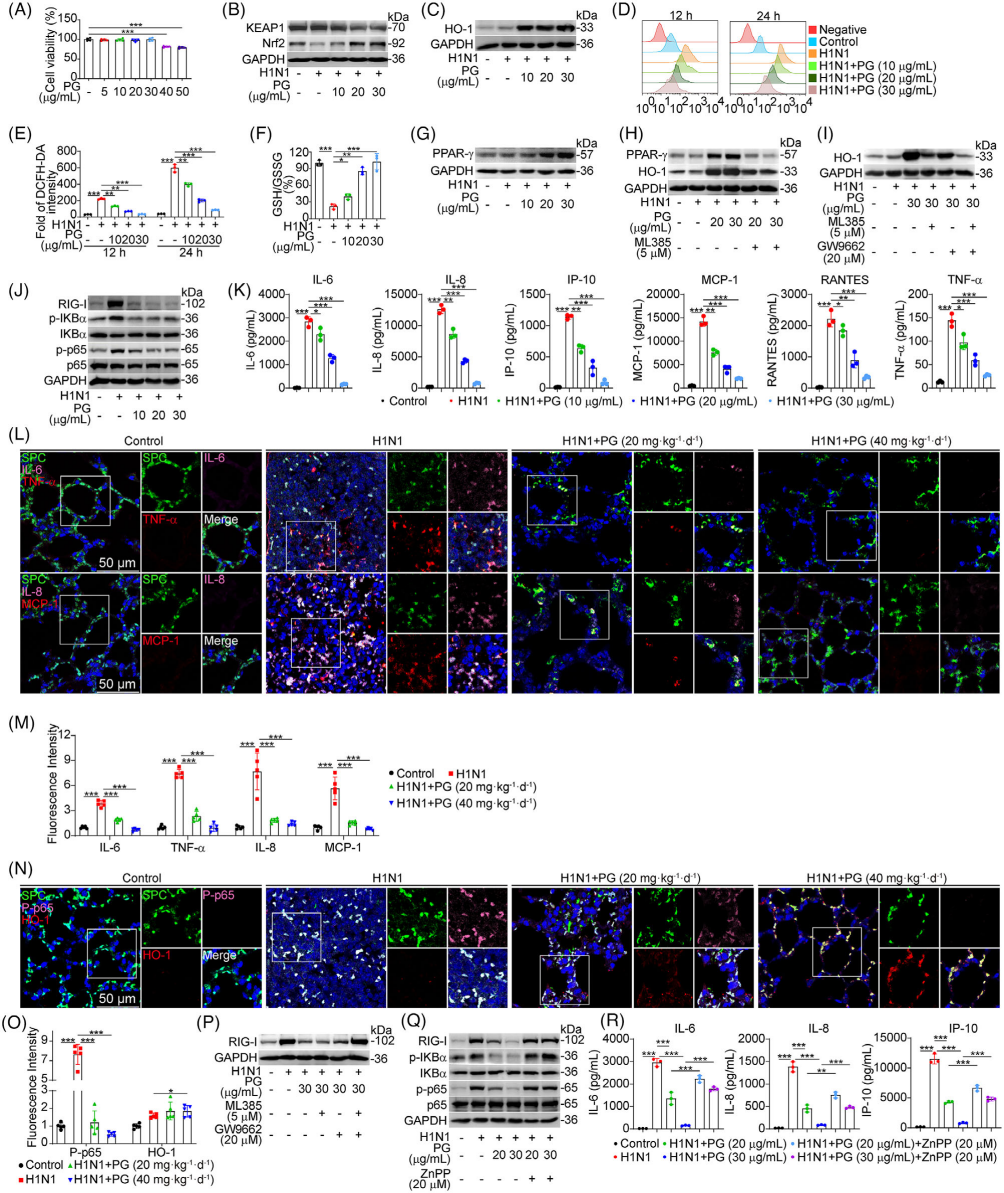
Effects of Nrf2–PPAR‐γ–HO‐1 signal axis inhibition on H1N1 virus‐activated RIG‐I–NF‐κB signaling in A549 cells with pyrogallol treatment. (A) The cytotoxicity of pyrogallol. (B) Immunoblot analysis of Nrf2 expression. (C) Immunoblot analysis of HO‐1 expression. (D and E) Levels of ROS. (F) GSH/GSSG ratio. (G) Immunoblot analysis of PPAR‐γ in A549 cells infected with the H1N1 virus and treated with pyrogallol. (H) Immunoblot analysis of PPAR‐γ and HO‐1 expression. (I) Immunoblot analysis of HO‐1 expression. (J) Immunoblot analysis of RIG‐I–NF‐κB signaling activation. (K) The Luminex assay was implemented to measure the levels of proinflammatory mediators (IL‐6, IL‐8, IP‐10, MCP‐1, RANTES, and TNF‐α) in the culture supernatant. (L) Four‐color immunofluorescence staining to measure the expression of proinflammatory mediators (IL‐6, TNF‐α, IL‐8, and MCP‐1) in SpC^+^ alveolar epithelial cells. (M) Panel representation of the relative fluorescence intensity of proinflammatory cytokines. Data were normalized to the control group. (N) Four‐color immunofluorescence staining to measure the expression of p‐p65 (pink) and HO‐1 (red) in SpC^+^ (green) alveolar epithelial cells. (O) Panel representation of the relative fluorescence intensity of p‐p65 and HO‐1. Data were normalized to the control group. (P) Expression of RIG‐I was examined by immunoblotting. (Q) Immunoblot analysis of RIG‐I, p‐IKBα, and p‐p65 expression. (R) Luminex assay was implemented to measure the levels of proinflammatory mediators (IL‐6, IL‐8, and IP‐10) in the culture supernatant. ^*^ *p* < 0.05, ^**^ *p* < 0.01, ^***^ *p* < 0.001.

MTT assay showed that pyrogallol, at a concentration ranging from 5 to 30 µg/mL, had no considerable impact on the viability of A549 cells (Figure 2A). Consequently, we chose a concentration of 30 µg/mL as the maximum concentration for subsequent experiments. Subsequently, we explored whether the protective effect of pyrogallol was correlated with the activation of Nrf2 signaling. As expected, we observed that pyrogallol treatment lowered the expression of KEAP1 (Figure 2B), which negatively regulates the Nrf2 pathway. The decrease in the levels of Nrf2 induced by H1N1 infection was effectively reversed by pyrogallol treatment (Figure 2B). Moreover, immunofluorescence staining indicated that treatment with pyrogallol caused the nuclear translocation of Nrf2 (Figure S1A). Moreover, the level of HO‐1, a downstream antioxidant protein of Nrf2, was significantly increased by pyrogallol (Figure 2C). The ROS level elevation induced by H1N1 virus infection was decreased by pyrogallol treatment (Figures 2D and E). And pyrogallol treatment was found to increase the GSH/GSSG ratio (Figure 2F). PPAR‐γ could also provide protective effects against ALI.^30^ Interestingly, our immunoblot analysis showed that pyrogallol treatment contributed to substantially enhanced expression of PPAR‐γ (Figure 2G) and promoted the nuclear translocation of PPAR‐γ (Figure S1B). Given that activation of Nrf2 signaling was implicated in reinforcing PPAR‐γ expression,^31^ we set out to clarify whether the increased expression of PPAR‐γ by pyrogallol was dependent on its activating effects on Nrf2 signaling. Indeed, the blockade of Nrf2 signaling by ML385 prominently reduced the pyrogallol‐induced increase in PPAR‐γ expression (Figure 2H). Interestingly, the inhibition of Nrf2 by ML385 did not completely abolish the increase in HO‐1 expression (Figure 2I), indicating that other factors activated by pyrogallol may also be linked to the elevation of HO‐1 expression. It was discovered that Nrf2 and PPAR‐γ collaborated to regulate HO‐1 expression.^31^ Figure 2I demonstrates that the combined treatment with ML385 and GW9662 led to lower HO‐1 levels in comparison with treatment with either inhibitor alone, indicating that the upregulation of Nrf2 and PPAR‐γ by pyrogallol jointly boosted the expression of HO‐1.

RIG‐I recognizes 5ʹ‐triphosphate‐modified RNA (5′PPP‐RNA, vRNA) derived from the invading viruses, contributing to sustained activation of NF‐κB signaling, resulting in H1N1 virus‐associated severe ALI.^16^, ^32^ We discovered that pyrogallol effectively inhibited the H1N1 virus‐mediated increase in RIG‐I levels, the overactivation of proteins associated with the NF‐κB pathway (p‐IKBα and p‐p65) (Figure 2J), and the nuclear translocation of p‐p65 (Figure S1C). Overproduction of NF‐κB‐dependent cytokines is believed to be detrimental during virus infection.^32^ As depicted in Figure 2K, pyrogallol treatment reduced the increased levels of proinflammatory mediators (IL‐6, IL‐8, IP‐10, MCP‐1, RANTES, and TNF‐α) induced by H1N1 virus infection (Figure 2K). Consistently, our coimmunofluorescence staining assay showed that pyrogallol administration effectively inhibited the H1N1 virus‐triggered enhancement of the expression of these proinflammatory mediators (IL‐6, TNF‐α, IL‐8, and MCP‐1) in alveolar epithelial cells (SpC^+^) (Figures 2L and M). Moreover, compared to H1N1 virus infection model mice, pyrogallol treatment decreased p‐p65 levels and enhanced HO‐1 levels in alveolar epithelial cells (SpC^+^) (Figures 2N and O). Existing research illustrates that Nrf2–PPAR‐γ–HO‐1 signaling activation can influence several cellular signals,^30^ including NF‐κB. Indeed, the expression level of RIG‐I after treatment with both Nrf2 and PPAR‐γ inhibitors was substantially higher relative to that observed in cells treated with a single inhibitor (Figure 2P). Additionally, the blockade of HO‐1 by ZnPP markedly abrogated the suppressive impact of pyrogallol on H1N1 virus‐activated RIG‐I–NF‐κB signaling molecules (RIG‐I, p‐IKBα, and p‐p65) (Figure 2Q). Next, it was found that HO‐1 inhibition reversed the suppressive impacts of pyrogallol on the H1N1 virus‐mediated increase in proinflammatory cytokines (IL‐6, IL‐8, and IP‐10) (Figure 2R). Moreover, pyrogallol treatment inhibited the increase in p‐IKBα and p‐p65 expression in H1N1 virus‐infected cells after transfection with a RIG‐I overexpression (OE) plasmid (Figure S1D), indicating that the inactivation of NF‐κB signaling by pyrogallol was due to suppression of RIG‐I‐mediated signal transmission, as upstream molecules of NF‐κB signaling. Meanwhile, transfection with vRNA, a ligand of RIG‐I, triggered the p‐IKBα and p‐p65 activation (Figure S1E), and the increased levels of proinflammatory mediators (IL‐6, IL‐8, IP‐10, and MCP‐1) (Figure S1F) were also suppressed by pyrogallol. Together, these data suggest that the upregulated expression of Nrf2 and PPAR‐γ by pyrogallol worked cooperatively to enhance HO‐1 expression, which led to abrogation of the aberrant activation of RIG‐I–NF‐κB signaling and thus decreased the excessive production of proinflammatory mediators.

### 2.3 Pyrogallol suppresses H1N1 virus‐mediated cell death by increasing HO‐1 expression

**FIGURE 3:**
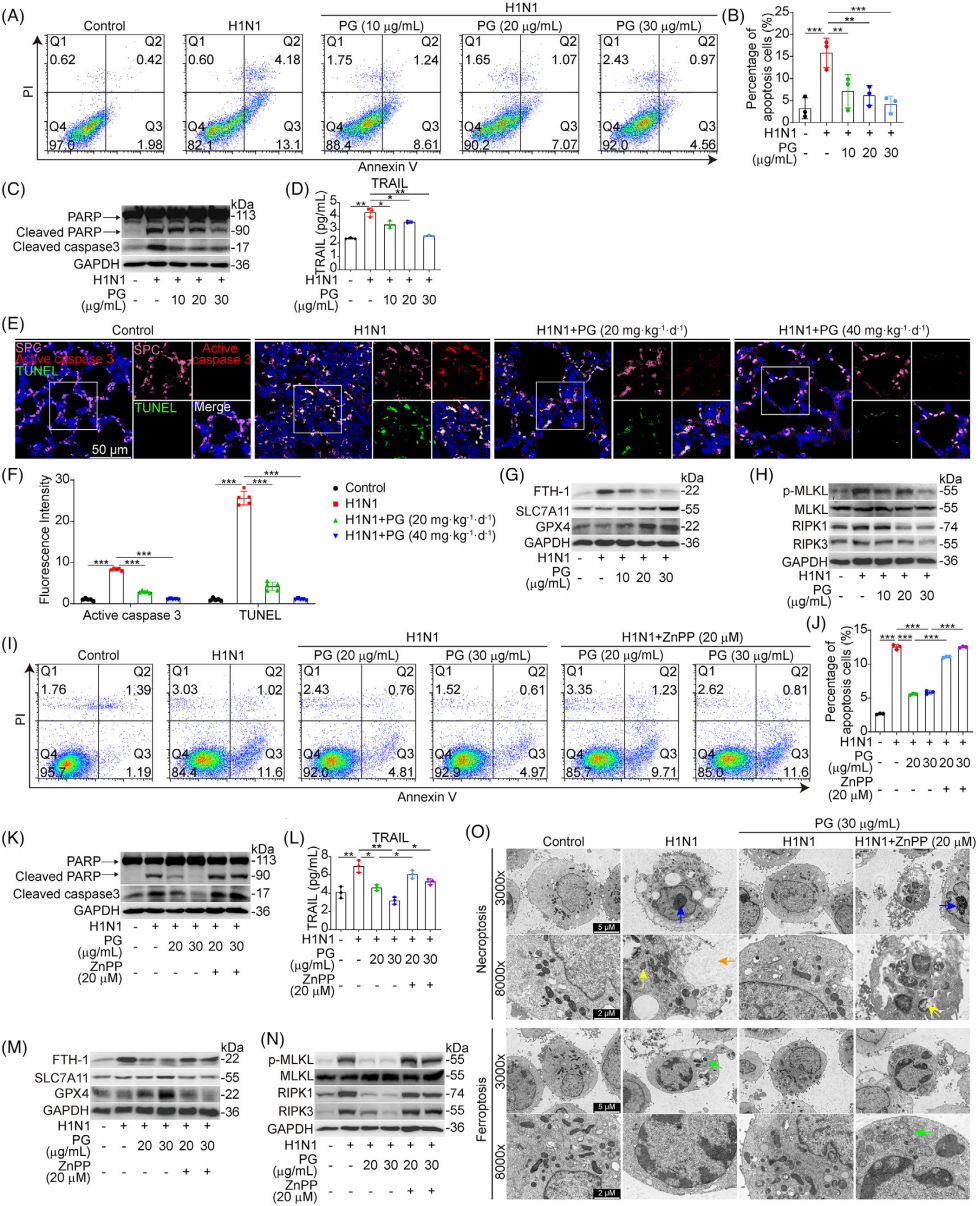
Effects of pyrogallol on H1N1 virus‐triggered apoptosis in vitro and in vivo. (A) Flow cytometry was used to identify the apoptotic state of A549 cells infected with the H1N1 virus. (B) The percentage of cells that have undergone apoptosis in panel (A). (C) Expression of cleaved caspase 3 and cleaved PARP as determined by immunoblotting. (D) Levels of TRAIL were measured by the Luminex assay. (E) Four‐color immunofluorescence staining to examine the apoptosis of SpC^+^ (pink) alveolar epithelial cells (red, active caspase 3; green, TUNEL) in the lungs. (F) Panel showing the relative fluorescence intensity of active caspase 3 and TUNEL. Data were normalized to the control group. (G) Immunoblot analysis of FTH‐1, SLC7A11, and GPX4 expression. (H) Immunoblot analysis of p‐MLKL, MLKL, RIPK1, and RIPK3 expression. (I) Flow cytometry was used to identify the apoptotic state of A549 cells infected with the H1N1 virus. (J) The percentage of apoptotic A549 cells infected with the H1N1 virus in panel (I). (K) Expression of cleaved caspase 3 and cleaved PARP as determined by immunoblotting. (L) The Luminex assay was used to assess the levels of TRAIL. (M) Immunoblot analysis of FTH‐1, SLC7A11, and GPX4 expression. (N) Immunoblot analysis of p‐MLKL, MLKL, RIPK1, and RIPK3 expression. (O) TEM analysis of the cellular ultrastructure. ^*^ *p* < 0.05, ^**^ *p* < 0.01, ^***^ *p* < 0.001.

The potential for cell death of alveolar epithelial cells induced by the influenza virus is another important component that might result in a fatal outcome.^27^ Therefore, we sought to find out whether the protective effects of pyrogallol against ALI were also related to the suppression of cell death in alveolar epithelial cells infected with the H1N1 virus. As illustrated in Figures 3A and B, A549 cells infected with the H1N1 virus showed a dose‐dependent reduction in the apoptosis level when treated with pyrogallol. The antiapoptotic properties of pyrogallol were validated by our immunoblotting results, which showed a decrease in apoptotic markers (cleaved caspase 3 and cleaved PARP) after pyrogallol treatment (Figure 3C). Moreover, we discovered that pyrogallol reduced the elevated TRAIL levels induced by the H1N1 virus (Figure 3D). Additionally, TUNEL staining and immunofluorescence staining demonstrated that the extensive apoptosis and elevated production of active caspase 3 in lung epithelial cells triggered by H1N1 virus were reduced by pyrogallol treatment (Figures 3E and F). Apart from apoptosis, cell death resulting from ferroptosis and necrosis could also contribute to H1N1 virus‐mediated severe ALI. As shown in Figure 3G, pyrogallol treatment downregulated FTH‐1 expression levels and upregulated SLC7A11 and GPX4 expression levels, indicating that H1N1 virus‐induced ferroptosis could be suppressed by pyrogallol. Moreover, the increased expression of necrosis‐related proteins (p‐MLKL, RIPK1, and RIPK3) induced by viral infection was reduced by pyrogallol (Figure 3H), suggesting that pyrogallol could alleviate H1N1 virus‐triggered necrosis. In addition to modulating proinflammatory reactions, Nrf2–PPAR‐γ–HO‐1 signaling also regulates cell death induced by various insults.^30^, ^33^ Our flow cytometry results showed that the blockade of HO‐1 markedly reversed the inhibition of H1N1 virus‐induced apoptosis by pyrogallol (Figure 3I and J). The reduction of apoptotic markers (cleaved PARP and cleaved caspase 3) by pyrogallol was also reversed by HO‐1 inhibition (Figure 3K). Simultaneously, the inhibition of H1N1 virus‐elevated TRAIL levels by pyrogallol was abolished by HO‐1 blockade (Figure 3L). Moreover, pyrogallol suppressed FTH‐1 expression and elevated SLC7A11 and GPX4 levels, whereas HO‐1 inhibition reversed these effects (Figure 3M). Pyrogallol inhibits necrosis‐related proteins (p‐MLKL, RIPK1, and RIPK3) (Figure 3N), whereas this effect is reversed when HO‐1 is blocked. The ultrastructure of H1N1 virus‐infected A549 cells treated with pyrogallol or combined with HO‐1 inhibitors was examined by transmission electron microscopy (TEM). Pyrogallol inhibited necroptosis (blue arrow: chromatin condensation; yellow arrow: mitochondrial swelling; orange arrow: plasma membrane rupture) and ferroptosis (green arrow: mitochondria shrinkage or mitochondrial crista breakage) in H1N1 virus infected cells, while the effect reversed by blocking HO‐1 (Figure 3O). These results point to a correlation between the inhibitory effects of pyrogallol on H1N1 virus‐induced cell death and its activation of Nrf2–PPAR‐γ–HO‐1 signaling.

### 2.4 Pyrogallol decreases the amplification of inflammation and apoptosis in cells with IFN‐β pretreatment prior to H1N1 virus infection

**FIGURE 4:**
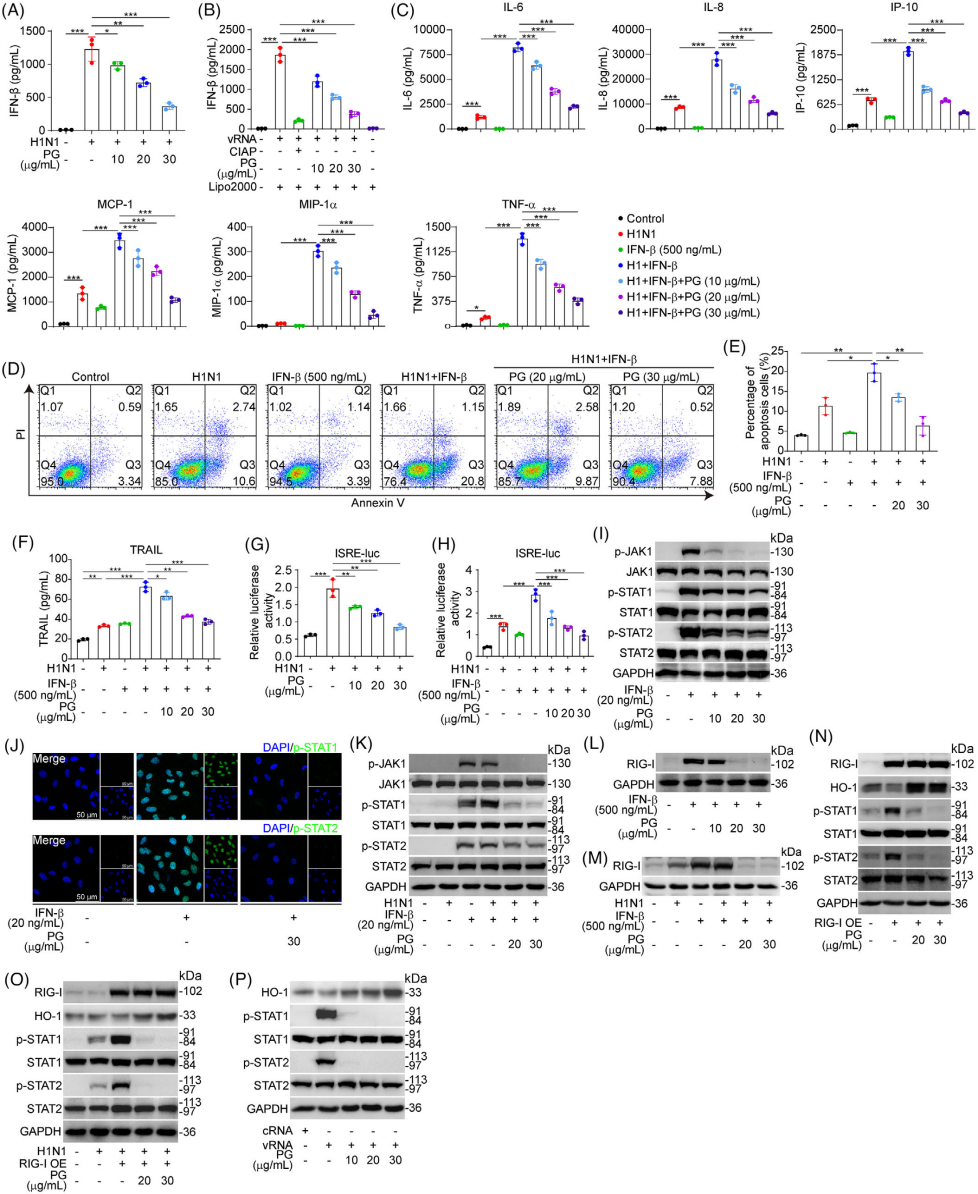
Effects of pyrogallol on H1N1 virus‐elicited amplification of the proinflammatory response in IFN‐β‐pretreated cells. (A and B) The concentration of IFN‐β in the cell culture supernatant of A549 cells infected with the H1N1 virus (A) and vRNA‐transfected A549 cells (B). (C) Luminex assay was implemented to measure the levels of proinflammatory mediators (IL‐6, IL‐8, IP‐10, MCP‐1, MIP‐1α, and TNF‐α) in the culture supernatant. (D) Flow cytometry was used to determine the apoptosis of H1N1 virus‐infected A549 cells that had been pretreated with IFN‐β. (E) The proportion of apoptotic cells in panel (D). (F) Levels of TRAIL were measured by Luminex assay. (G) The ISRE‐luciferase reporter activity was measured in H1N1 virus‐infected A549 cells. (H) ISRE‐luciferase reporter activity in A549 cells pretreated with IFN‐β (500 ng/mL) before H1N1 virus infection. (I) Immunoblotting of p‐JAK1, p‐STAT1, and p‐STAT2 expression in A549 cells stimulated for 15 min with IFN‐β (20 ng/mL). (J) Immunofluorescence analysis was conducted to detect the nuclear localization of p‐STAT1 and p‐STAT2 in A549 cells stimulated for 15 min with IFN‐β (20 ng/mL). (K) Immunoblotting of p‐JAK1, p‐STAT1, and p‐STAT2 expression in A549 cells infected with H1N1 virus for 4 h followed by 15 min of stimulation with IFN‐β (20 ng/mL). (L) The expression of RIG‐I in A549 cells was examined by immunoblotting after 24 h of stimulation with IFN‐β (500 ng/mL). (M) Expression of RIG‐I in A549 cells treated with IFN‐β (500 ng/mL) for 4 h before H1N1 virus infection was examined by immunoblotting. (N) A549 cells transfected with the RIG‐IOE plasmid were analyzed by immunoblotting for the expression of p‐STAT1 and p‐STAT2. (O) H1N1 virus infection of RIG‐I OE plasmid‐transfected A549 cells for 8 h was analyzed by immunoblotting for p‐STAT1 and p‐STAT2 expression. (P) Immunoblot analysis of p‐STAT1 and p‐STAT2 expression in A549 cells with vRNA transfection. ^*^ *p* < 0.05, ^**^ *p* < 0.01, ^***^ *p* < 0.001.

Influenza virus‐triggered RIG‐I signaling cascade activation enhanced IFN‐β expression, which in turn exacerbated the proinflammatory response and ALI.^28^ Pyrogallol treatment resulted in a significant reduction in the expression of IFN‐β in A549 cells that were infected with the H1N1 virus (Figure 4A). A similar pattern was seen for IFN‐β expression in cells transfected with influenza virus‐derived vRNA, a potent inducer of IFN‐β (Figure 4B). Next, we found that the expression of proinflammatory mediators (IL‐6, IL‐8, IP‐10, MCP‐1, MIP‐1α, and TNF‐α) was substantially increased in cells that had been stimulated with IFN‐β before the infection with the H1N1 virus. However, the levels of these mediators were remarkably decreased by pyrogallol (Figure 4C). Along with the amplification of proinflammatory reactions, prestimulation with IFN‐β has been reported to promote the expression of the apoptosis factor TRAIL.^34^ Apoptotic activity was shown to be greater in cells that had been prestimulated with IFN‐β before H1N1 viral infection in comparison with cells that had been treated with IFN‐β or H1N1 virus alone; however, pyrogallol treatment was able to reduce apoptotic activity (Figures 4D and E). Similarly, pyrogallol was shown to inhibit the increased expression of TRAIL (Figure 4F). We postulated that the inhibition of ISGF3 transcriptional activity was responsible for the suppressive impact of pyrogallol on the IFN‐β‐induced amplification of the proinflammatory response and apoptotic activity induced by the H1N1 virus. As expected, treating cells transfected with the ISRE‐luciferase reporter plasmid and infected with the H1N1 virus with pyrogallol reduced the enhanced transcriptional activity of ISGF3 (Figure 4G). Furthermore, ISGF3 transcriptional activity was higher in cells that had been prestimulated with IFN‐β before to viral infection than in cells infected with the virus alone; pyrogallol was also able to suppress this activity (Figure 4H).

We conducted immunoblotting to detect JAK1, STAT1, and STAT2 activation to help clarify whether the suppressive impact of pyrogallol on the enhanced proinflammatory response was linked to the formation of the ISGF3 complex. Figure 4I showed that the IFN‐β‐induced activation of JAK1 and the downstream STATs were effectively abolished in cells pretreated with pyrogallol for 12 h. Pyrogallol blocked the nuclear translocation of STAT1 and STAT2 (Figure 4J). Similar to IFN‐β stimulation alone, JAK1, STAT1, and STAT2 activation mediated by the combined IFN‐β and H1N1 virus stimulation was inhibited by pyrogallol treatment (Figure 4K). Interestingly, we discovered that the elevated expression levels of RIG‐I by IFN‐β stimulation were downregulated by pyrogallol (Figure 4L). Moreover, A549 cells treated with both IFN‐β and H1N1 virus were found to have elevated levels of RIG‐I in comparison to cells treated with IFN‐β or H1N1 virus stimulation alone, and pyrogallol effectively reduced these levels (Figure 4M). Research has shown that RIG‐I can activate STAT1.^35^ A plasmid overexpressing RIG‐I was shown to enhance STAT1 and STAT2 activation in transfected cells, whereas pyrogallol treatment decreased this activation (Figure 4N). Moreover, pyrogallol suppressed the increase in STAT1 and STAT2 activation in H1N1 virus‐infected cells transfected with a plasmid overexpressing RIG‐I (Figure 4O). Meanwhile, in cells transfected with vRNA, the activation of STAT1 and STAT2 was triggered, which was inhibited by pyrogallol (Figure 4P). Collectively, these data indicate that pyrogallol diminished the amplification of the H1N1 virus‐induced proinflammatory reaction in IFN‐β‐sensitized cells, which resulted from the inhibition of JAK1 activation and RIG‐I expression, abrogating STAT1 and STAT2 phosphorylation and thus blocking ISGF3 transcriptional activity.

### 2.5 Blockade of HO‐1 diminishes the suppressive effects of pyrogallol on the enhancement of the inflammation and apoptosis in cells with IFN‐β pretreatment prior to H1N1 virus infection

**FIGURE 5:**
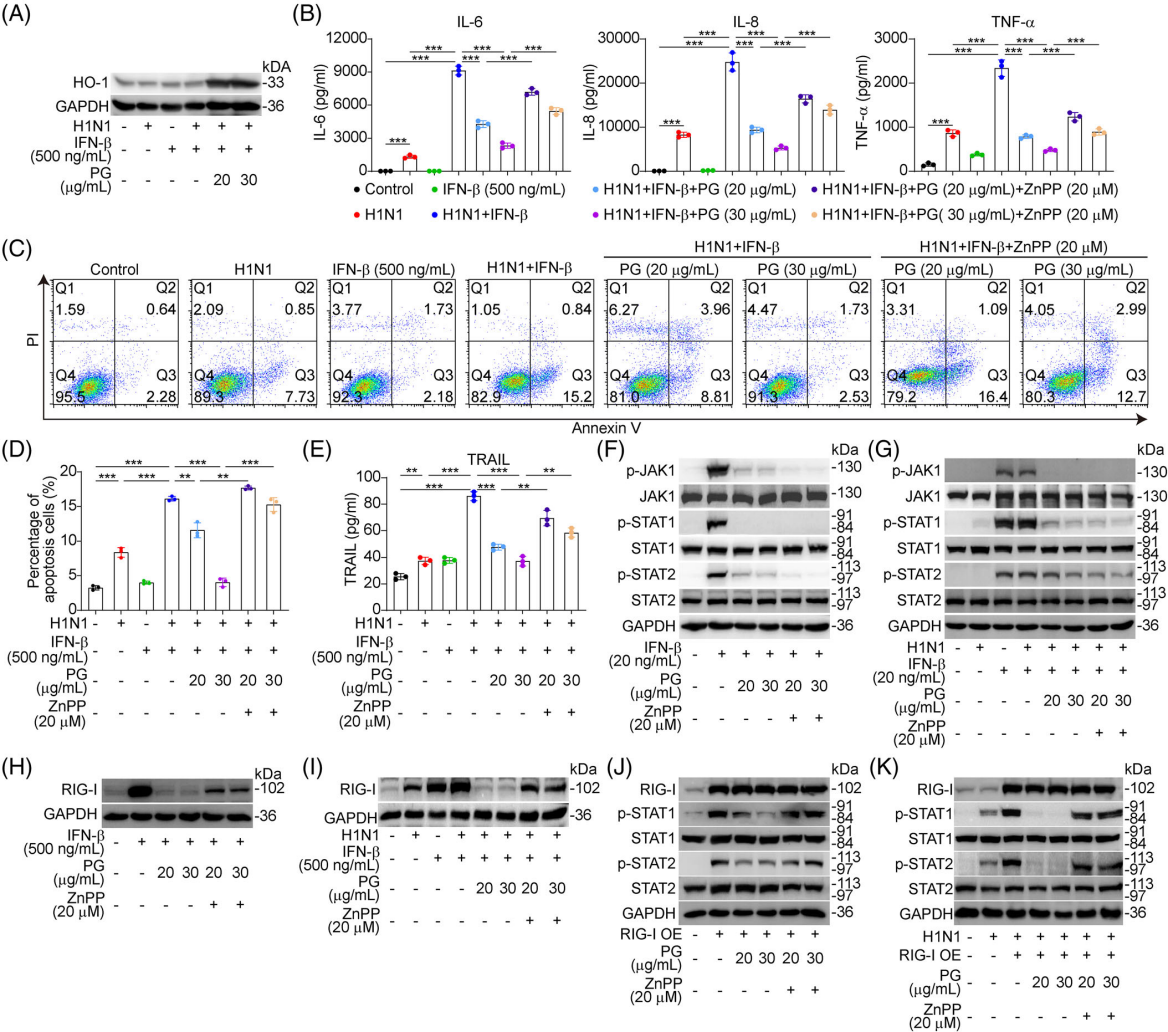
Involvement of HO‐1 in the inhibitory effect of pyrogallol on IFN‐β‐mediated amplification of the proinflammatory response and exacerbated apoptosis in cells with H1N1 virus infection. (A) Expression of HO‐1 in A549 cells treated with IFN‐β (500 ng/mL) for 4 h before H1N1 virus infection was examined by immunoblotting. (B) Levels of proinflammatory mediators (IL‐6, TNF‐α, and IP‐10) in A549 cells stimulated with IFN‐β (500 ng/mL) for 4 h before H1N1 virus infection. (C) Flow cytometry was used to assess the apoptosis of H1N1 virus‐infected A549 cells that had been pretreated with IFN‐β. (D) The proportion of apoptotic cells in panel (C). (E) The levels of TRAIL were measured by the Luminex assay. (F) Expression of p‐JAK1, p‐STAT1, and p‐STAT2 in A549 cells treated with IFN‐β (20 ng/mL) for 15 min was analyzed by immunoblotting. (G) Expression of p‐JAK1, p‐STAT1, and p‐STAT2 in A549 cells stimulated with IFN‐β (20 ng/mL) for 15 min after infection with H1N1 virus for 4 h was analyzed by immunoblotting. (H) Analysis of the expression of RIG‐I by immunoblotting in A549 cells treated with IFN‐β (500 ng/mL) for 24 h. (I) Evaluation of RIG‐I expression by immunoblotting in A549 cells treated with IFN‐β (500 ng/mL) for 4 h before infection with the H1N1 virus. (J) Assessment of p‐STAT1 and p‐STAT2 expression in A549 cells transfected with the RIG‐I OE plasmid using immunoblotting. (K) Expression of p‐STAT1 and p‐STAT2 in A549 cells transfected with the RIG‐I OE plasmid and infected for 8 h with the H1N1 virus was analyzed by immunoblotting. ^*^ *p* < 0.05, ^**^ *p* < 0.01, ^***^ *p* < 0.001.

In cells that had been pretreated with IFN‐β before viral infection, our immunoblotting analysis revealed that pyrogallol treatment enhanced HO‐1 expression (Figure 5A). In contrast, inhibiting HO‐1 reversed the suppressive impacts of pyrogallol on IFN‐β‐triggered amplification of proinflammatory cytokines in cells infected with H1N1 virus (Figure 5B). Moreover, HO‐1 inhibition also abolished the suppressive impacts of pyrogallol on the enhanced apoptosis (Figures 5C and D) and the increased expression of TRAIL (Figure 5E) in H1N1 virus‐infected cells prestimulated with IFN‐β. However, we found that blocking HO‐1 had no reversal impact on the inhibitory impacts of pyrogallol on IFN‐β‐mediated phosphorylation of JAK1 and the downstream STATs (Figure 5F). Similar results were obtained when cells were sensitized by IFN‐β before viral infection (Figure 5G). These data illustrated that the inhibitory impact of pyrogallol on IFN‐β‐induced phosphorylation of JAK1, STAT1, and STAT2 was not dependent on increased HO‐1 levels. Furthermore, we found that the inhibitory effects of pyrogallol on IFN‐β‐induced RIG‐I were reversed by HO‐1 inhibition (Figure 5H). Meanwhile, compared with cells stimulated with IFN‐β or infected with viruses alone, the higher levels of RIG‐I in cells exposed to both IFN‐β and viruses were decreased by pyrogallol, which was abolished by HO‐1 inhibition (Figure 5I). The inhibitory impact of pyrogallol on the STAT1 and STAT2 activation in A549 cells with a plasmid overexpression RIG‐I was found to be diminished upon HO‐I inhibition (Figure 5J). Likewise, the inhibitory impact of pyrogallol on the enhanced activation of STAT1 and STAT2 in H1N1 virus‐infected cells transfected with the plasmid overexpressing RIG‐I was reversed by HO‐I inhibition (Figure 5K). Collectively, these results suggest that pyrogallol reduced the enhancement of the proinflammatory reaction and apoptotic activity that was mediated by IFN‐β in H1N1 virus‐infected cells, which is attributed to the HO‐1‐dependent inhibition of RIG‐I‐augmented STAT1 activation and the HO‐1‐independent inactivation of JAK1.

### 2.6 Inhibition of HO‐1 abolishes the protective effects of pyrogallol against H1N1 virus‐mediated ALI

**FIGURE 6:**
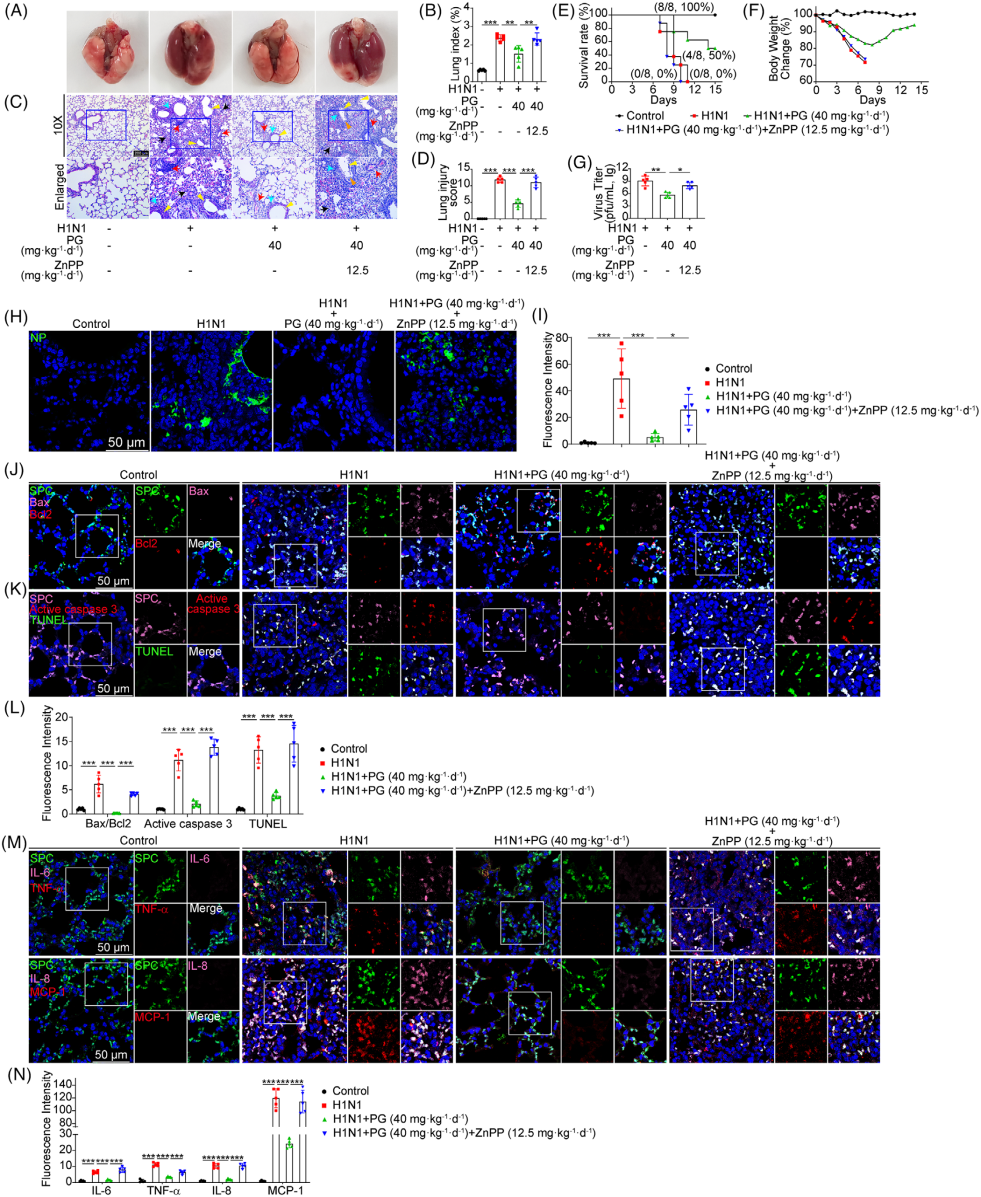
Effects of HO‐1 inhibition on the protective effects of pyrogallol against H1N1 virus‐mediated ALI. (A) Gross anatomy of the H1N1 virus‐infected lungs. (B) Lung index (lung/body weight ratio). (C) H&E staining of lung tissues (×100, scale bar = 200 µm). Black arrows: alveolitis; red arrows: lung vasculitis; blue arrows: inflammation around the bronchioles; orange triangle: bronchiolitis; yellow triangle: bronchiolar epithelial sloughing. (D) The lung injury was scored. (E and F) Kaplan–Meier survival curve (E) and changes in the body weight (F) in mouse‐adapted A/FM/1/47(H1N1)‐infected mice (*n* = 8 mice per group). (G) Viral titers in the lung tissues. (H) Immunofluorescence staining for measuring the expression of NP (green). (I) NP's relative intensity of fluorescence. (J) Four‐color immunofluorescence staining to assess the expression of Bax (pink) and Bcl2 (red) in SpC^+^ (green) alveolar epithelial cells. The quantification of the relative fluorescence intensities of Bax/Bcl2 was performed in panel (L). Data were normalized to the control group. (K) Four‐color immunofluorescence staining for determining the apoptosis of SpC^+^ (pink) alveolar epithelial cells (red, active caspase 3; green, TUNEL) in the lungs. (L) The relative fluorescence intensities of active caspase 3 and TUNEL in panel (K) were quantified. Data were normalized to the control group. (M) Four‐color immunofluorescence staining for measuring the expression of proinflammatory cytokines (IL‐6, TNF‐α, IL‐8, and MCP‐1) in SpC^+^ alveolar epithelial cells. (N) The relative fluorescence intensities of proinflammatory cytokines (IL‐6, TNF‐α, IL‐8, and MCP‐1) were quantified. Data were normalized to the control group. ^*^ *p* < 0.05, ^**^ *p* < 0.01, ^***^ *p* < 0.001.

To determine whether the protective function of pyrogallol against H1N1 virus‐mediated ALI is dependent on HO‐1 upregulation, we performed in vivo experiments. Anatomic pathology examination of the lungs revealed that the alleviation of H1N1 virus‐elicited lung edema and bleeding by pyrogallol administration was abrogated by HO‐1 inhibition (Figure 6A). HO‐1 inhibition diminished the reduction of the increased lung index, a parameter reflecting lung injury, by pyrogallol (Figure 6B). Meanwhile, a histological analysis of the lungs revealed that pyrogallol alleviated the destruction of lung tissues induced by the H1N1 virus, including alveolar collapse, proinflammatory cell lung infiltration, bronchiolitis, and vasculitis, which were also weakened upon HO‐1 inhibition (Figure 6C). Accordingly, pathological scores showed that intraperitoneal administration of ZnPP attenuated the protective properties of pyrogallol against ALI in mice infected with the H1N1 virus (Figure 6D). The survival benefits of pyrogallol treatment (40 mg·kg^−1^·day^−1^) in H1N1 virus‐infected mice were found to be abolished in mice treated with a combination of pyrogallol and ZnPP (Figure 6E). The body weight changes (Figure 6F), viral titers (Figure 6G), and the expression of NP (Figures 6H and I) in the lung tissues showed a similar tendency. Furthermore, coimmunofluorescence staining was performed to investigate whether HO‐1 inhibition affected the antiapoptotic and anti‐inflammatory activities of pyrogallol in vivo. As illustrated in Figures 6J and L, blocking HO‐1 significantly reversed the suppressive impact of pyrogallol on the proapoptotic protein Bax as well as the enhancing effects of pyrogallol on the antiapoptotic protein Bcl2 in alveolar epithelial cells (SpC^+^). Meanwhile, TUNEL staining and immunofluorescence assays showed that HO‐1 inhibition abolished the suppressive impact of pyrogallol on H1N1 virus‐induced apoptosis and the elevated production of active caspase 3 in lung epithelial cells (Figures 6K and L). Furthermore, a comparable pattern was observed for the proinflammatory cytokine levels (IL‐6, TNF‐α, IL‐8, and MCP‐1) of alveolar epithelial cells (SpC^+^) in mice treated with ZnPP and pyrogallol (Figures 6M and N). Taken together, our research demonstrates that pyrogallol protects against ALI induced by the H1N1 virus via upregulation of HO‐1 in vivo.

## 3 DISCUSSION

**FIGURE 7:**
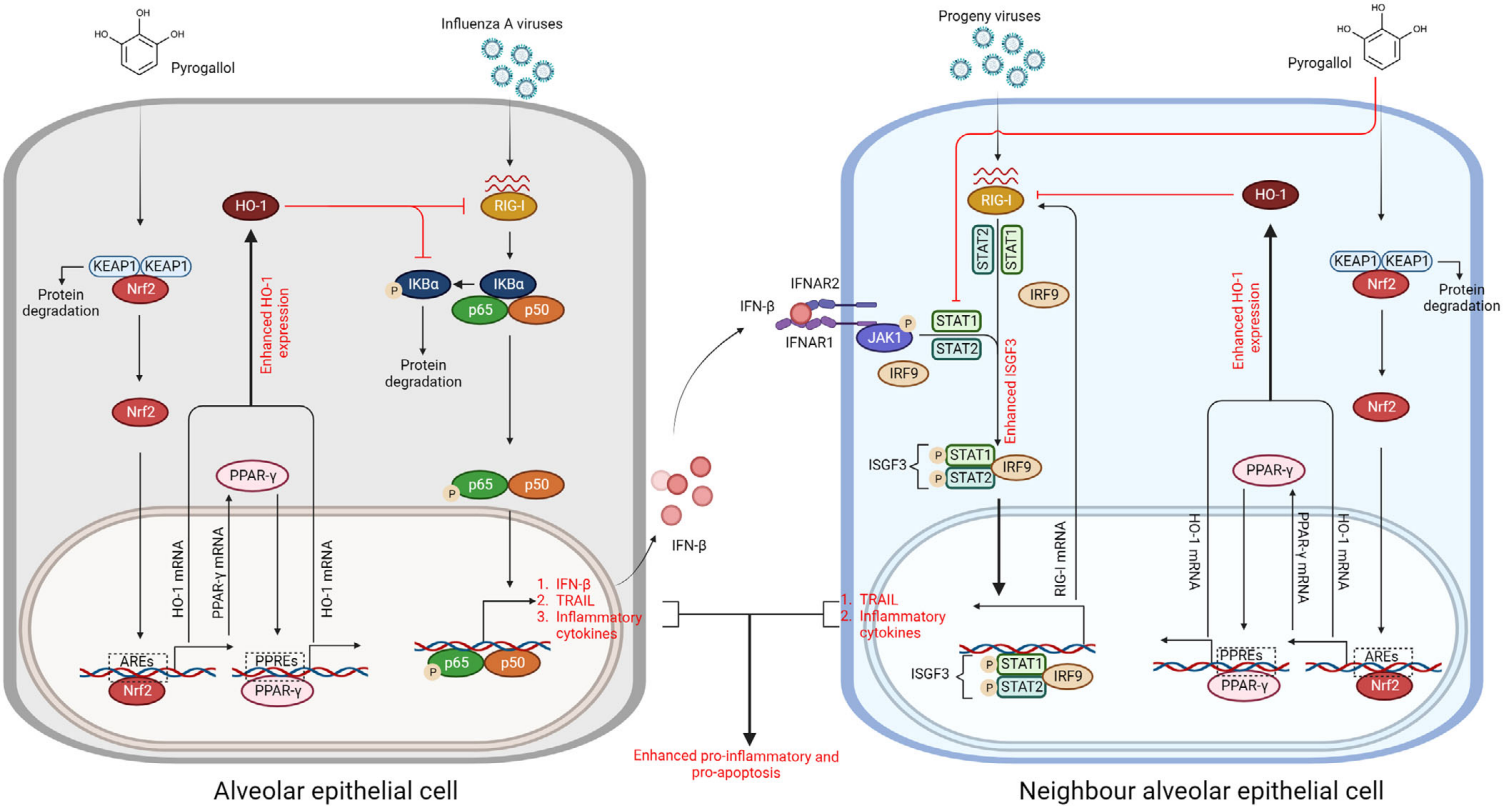
Schematic diagram depicting the underlying mechanism by which pyrogallol confers protection against excessive inflammation and ALI induced by the H1N1 virus.

In this study, we found that pyrogallol protected against H1N1 virus‐mediated ALI, resulting from activation of both Nrf2 and PPAR‐γ, which acted synergistically to enhance HO‐1 production; this further exerted inhibitory effects on RIG‐I–NF‐κB signaling and the enhanced transcriptional activity of ISGF3 via the activating effect of RIG‐I on STAT1/2, contributing to the inhibition of the proinflammatory reactions and cell death (Figure 7). Together, our data provided new insight into the mechanism of pyrogallol in treatment of influenza‐associated lung injury.

Enhanced activation of RIG‐I–NF‐κB signaling in patients infected with the highly pathogenic avian H5N1 virus can result in life‐threatening cytokine storm syndrome.^22^ Based on findings obtained from in vitro and in vivo investigations, pyrogallol was shown to be efficacious in inhibiting the activation of RIG‐I–NF‐κB signaling and the expression of proinflammatory cytokines (IL‐8, TNF‐α, IL‐6, and MCP‐1) that were induced by the H1N1 virus. In vitro and in vivo investigations have shown evidence that pyrogallol suppresses the activation of RIG‐I–NF‐κB signaling induced by the H1N1 virus as well as the expression levels of proinflammatory cytokines (MCP‐1, IL‐8, TNF‐α, and IL‐6). Moreover, we observed that pyrogallol treatment improved the H1N1 virus‐induced increased lung index, lung damage, and high mortality in mice. Individuals with influenza infection rapidly progress to severe ALI or ARDS, which often results from endothelial barrier breakdown with liquid and protein crossing the barrier into alveoli and/or lung epithelial sodium channel dysfunction with lung water retention.^36^, ^37^ Furthermore, we found that pyrogallol robustly upregulated the HO‐1 levels. Elevation of HO‐1 levels has been found to suppress influenza virus‐induced RIG‐I signaling cascade activation, including the JNK‐MAPK and NF‐κB pathways.^38^, ^39^ HO‐1 exerts its biological activity via the catabolism of heme into ferrous iron (Fe^2+^), biliverdin IXα, and carbon monoxide (CO).^40^ There is evidence that administration of CO, biliverdin IXα, and ferrous iron reduces excessive inflammation and ALI via inhibition of NF‐κB signaling.^40^, ^41^ The HO‐1 blockade was shown to reverse the suppressive impact of pyrogallol on H1N1 virus‐mediated RIG‐I–NF‐κB signaling, proinflammatory reactions, and ALI, indicating pyrogallol exerts anti‐inflammatory effects in an HO‐1‐dependent manner. Based on these observations, we suppose that the protective effects of pyrogallol against lethal viral infection are closely associated with elevated levels of HO‐1, which exerts suppressive effects on RIG‐I–NF‐κB‐mediated excessive inflammation.

Apart from excessive proinflammatory mediators, the production of type I IFNs mediated by RIG‐I–NF‐κB signaling exerted a detrimental role in infectious illnesses.^26^, ^27^ It was found that blockade of type I IFN signaling alleviates lethal SARS‐CoV‐ and influenza virus‐mediated morbidity and mortality.^26^, ^42^ Surprisingly, the elevation of type I IFN levels triggered by the influenza virus promotes the development of secondary bacterial pneumonia.^43^ The antiviral actions of type I IFNs were found to be counteracted by viruses, but excessive inflammation is elicited by type I IFNs, which is believed to contribute to the pathogenic effects of type I IFNs in viral infection.^44^ Therefore, we speculate that the suppressive effects of pyrogallol on the IFN‐β‐triggered amplification of proinflammatory responses facilitate the prevention of H1N1 virus‐mediated severe ALI. Previous studies found that amplification of proinflammatory reactions by type I IFN signaling resulted in H1N1 virus‐mediated severe ALI.^28^, ^29^ Our results showed that HO‐1 inhibition abolished the inhibitory impact of pyrogallol on the activation of STAT1 and STAT2 in cells with RIG‐I overexpression, the upregulation of RIG‐I by IFN‐β, and the enhanced expression of RIG‐I in H1N1 virus‐infected cells pretreated with IFN‐β. Existing literature has provided evidence that increased levels of HO‐1 inactivate STAT1 in response to LPS stimulation or viral infection.^45^, ^46^ A previous study suggested that the induction of RIG‐I by IFN‐β has a converse effect on the activation of STAT1.^35^ Moreover, our findings reveal that not only STAT1 but also another component of the ISGF3 complex, STAT2, could be activated in response to RIG‐I overexpression, which improves our understanding of the enhanced proinflammatory response and the reinforced transcriptional activity of ISGF3 in IFN‐β‐sensitized cells. Moreover, HO‐1 blockade could not reverse the suppressive effects of pyrogallol on IFN‐β‐induced JAK1 activation. These results collectively suggest that pyrogallol can suppress IFN‐β‐induced JAK1 activation and elevate HO‐1 expression, resulting in STAT1/2 inactivation, which in turn reduces the transcriptional activity of ISGF3 and the amplification of H1N1 virus‐mediated proinflammatory processes triggered by IFN‐β pretreatment.

Additionally, aberrant alveolar epithelial cell death is a significant contributor to the severity of influenza infections.^26^, ^27^, ^47^ Increased cell death in virus‐infected and noninfected alveolar epithelial cells substantially impairs gas exchange, resulting in patients with influenza illness progressing to ARDS.^27^ In vitro and in vivo tests demonstrated that treatment of H1N1 virus‐infected cells with pyrogallol decreased the increase in cell death. In addition to proinflammatory mediators and IFNs, activation of RIG‐I–NF‐κB signaling is involved in driving the expression of apoptotic factors (TRAIL and FasL), promoting apoptosis of influenza virus‐infected cells.^48^ Hence, we hypothesize that the antiapoptotic impacts of pyrogallol against ALI induced by the H1N1 virus might be closely related to the inhibition of RIG‐I–NF‐κB signaling. Moreover, we provided evidence that the inhibitory impact of pyrogallol on virus‐induced cell death was linked to its enhancing effects on HO‐1 expression and that blockade of HO‐1 reversed those effects. Accumulating evidence confirmed the anticell death properties (including apoptosis, ferroptosis, and necroptosis) of HO‐1 and the products of heme metabolism (e.g., CO) in the situation of ALI triggered by various insults.^49^, ^50^, ^51^ In addition to RIG‐I–NF‐κB signaling, it was shown that IFN‐β sensitization promoted the expression of TRAIL and apoptosis of alveolar epithelial cells infected with the H1N1 virus, which was prominently abolished by pyrogallol. Moreover, we found that inhibition of HO‐1 reversed the inhibitory impact of pyrogallol on the IFN‐β‐triggered increase in TRAIL expression and apoptosis in cells infected with the H1N1 virus. Research has revealed that the ISRE sequence within the promoter region of TRAIL was bound by type I IFN‐activated ISGF3, which in turn drove the expression of TRAIL and apoptosis.^34^, ^52^ Similarly, increased levels of TRAIL in mice infected with the influenza virus resulted in significant lung damage, and ARDS was discovered to be type I and II IFN‐dependent.^27^, ^53^ For the STAT1/2 inactivation properties of HO‐1, we suppose that the weakening of the antiapoptotic effects of pyrogallol by HO‐1 blockade in IFN‐β‐sensitized cells was due to a diminished capacity for HO‐1‐dependent inhibition of STAT1/2 activation, leading to impaired inhibitory effects on the transcriptional activity of ISGF3 and thereby abolishing the reducing effects on the expression of TRAIL. Together, our data suggested that pyrogallol could be a promising agent for reducing excessive inflammation and aberrant cell death in the treatment of influenza infection.

## 4 MATERIALS AND METHODS

### 4.1 Reagents and antibodies

Pyrogallol (purity > 99.98%; HY‐N1579) (Figure 1A) and ML385 (HY‐100523) were obtained from MedChemExpress. The HO‐1 inhibitor ZnPP was purchased from AdooQ BioScience (Nanjing, China). MTT was purchased from Sigma‐Aldrich. DAPI (C1006) was obtained from Beyotime Biotechnology, Inc. (Shanghai, China). Recombinant human IFN‐β (#300‐02BC) was produced by Peprotech, Inc. (Rocky Hill, NJ, USA). Bead‐based multianalyte profiling kits for quantification of MIP‐1α, TNF‐α, MCP‐1, IL‐8, IP‐10, TRAIL, IL‐6, and RANTES were procured from Bio‐Rad Laboratories Inc. (Hercules, CA, USA). Antibodies are shown in Table S1.

### 4.2 Cell lines, viruses, and viral infection

DMEM/F12 with 10% (v/v) fetal bovine serum was used to culture human A549 alveolar epithelial cells (ATCC; CCL‐185) at 37°C in a humidified environment with 5% CO_2_.

Influenza viruses, including A/PR/8/34 (H1N1), A/GZ/GIRD07/09 (H1N1), A/HK/8/68 (H3N2), A/HK/Y280/97(H9N2), A/Duck/Guangdong/1994 (H7N3) and mouse‐adapted virus A/FM/1/47(H1N1) were obtained from the State Key Laboratory of Respiratory Disease and stored at −80°C. All viral strains were propagated in the allantoic cavities of 10‐day‐old embryonated chicken eggs and virus titers were determined by titration on MDCK cells as previously described.^54^

### 4.3 MTT assay

After the indicated treatment, the culture medium of cells was replaced with 100 µL of MTT solution (5 mg/mL). Following a further 4‐h incubation period, 200 µL of dimethyl sulfoxide was used to dissolve the water‐insoluble formazan crystals and a microplate reader (Thermo Fisher Scientific, Waltham, MA, USA) was utilized to measure the optical density values at 570 nm.

### 4.4 Luminex assay

Cellular debris was removed by centrifuging cell culture supernatants at 4°C for 15 min at 16,200 g. Then, the supernatants were aliquoted and immediately preserved at −80°C before analysis. The levels of chemokines and cytokines in the supernatants were determined using Bio‐Rad cytokine assay kits in line with the guidance provided by the manufacturer.

### 4.5 Western blot analysis

A bicinchoninic acid protein assay kit (Thermo Fisher Scientific) was utilized to measure the protein concentrations in whole cell lysates. Next, SDS‐PAGE (20 µg per lane) was used to separate the proteins before blotting them onto 0.2 µm PVDF membranes. The membranes were subjected to incubation with primary antibodies for one night after being blocked with 5% nonfat milk. The next step was to incubate the membranes for 1 h with secondary antibodies that were conjugated to HRP. An enhanced chemiluminescence reaction kit (Amersham Biosciences) was used to detect protein bands.

### 4.6 Immunofluorescence assay

Methanol was used to fix the cells for 15 min. After 15 min of permeabilization with 0.5% Triton X‐100 in PBS, the cells were blocked for half an hour with goat serum. Then, coverslips were subjected to incubation with the designated primary antibodies for a whole night at 4°C, followed by a wash and an hour‐long incubation with secondary antibodies conjugated with FITC. Thereafter, DAPI (1 µg/mL) was used to stain the nuclei for 10 min. Finally, images were captured using a Zeiss Axiovert 135 fluorescence microscope.

### 4.7 Transmission electron microscopy

At 24 h p.i., H1N1 virus‐infected cells were harvested and fixed in 2.5% cold glutaraldehyde (G1102; Servicebio, Wuhan, China). Ultrathin sections were prepared and analyzed using a HT7700 TEM (Hitachi, Tokyo, Japan) as previously described.^55^

### 4.8 Animal experiments

Female C57BL/6 mice (weighing 16−20 g at 4−6 weeks of age) were purchased from Guangdong Medical Laboratory Animal Center (Guangzhou, China). Animals were housed in a specific pathogen‐free environment with unrestricted access to water and food, as well as regulated light cycle (12/12‐h light/dark cycle) and temperature. The following four groups of mice were established at random: (i) the normal group; (ii) the H1N1 virus infection group, in which untreated mice were infected with 5× LD_50_ of mouse‐adapted A/FM/1/47(H1N1) influenza virus; and (iii and iv) the H1N1 + pyrogallol groups, in which H1N1‐infected mice received pyrogallol (20 or 40 mg·kg^−1^·day^−1^ for 7 days) intragastrically until 2 days before viral infection.

### 4.9 Histologic analysis, lung injury score, and coimmunofluorescence assay

To stain the lung tissues, they were cut into 4‐µm‐thick sections and then subjected to H&E staining. The lung injury was scored by two experienced independent blinded researchers, and the scoring criteria were applied as previously described.^56^ A TSAPLus fluorescent triple staining kit (G1236‐100T; Servicebio) was applied to label the indicated antigens as directed by the manufacturer. The apoptotic signals were labeled with the help of the One‐step TUNEL In Situ Apoptosis Kit (Green, FITC) (E‐CK‐A320; Elabscience Biotechnology Co., Ltd., Wuhan, China).

### 4.10 Apoptosis assay

Cells in six‐well plates were harvested and resuspended in 1× binding buffer (100 µL) at a density of 2 × 10^6^ cells/mL. Thereafter, the cell suspensions were stained with Annexin V‐FITC (2.5 µg/mL) and propidium iodide (50 µg/mL) and incubated for 15 min in darkness at room temperature. Next, a FACScan flow cytometer (Accuri C6; BD Biosciences) was utilized to analyze the cells.

### 4.11 Reporter plasmid transfection and luciferase assay

Beyotime (Shanghai, China) supplied the ISRE firefly luciferase reporter plasmid. The cells were cotransfected with two plasmids: the ISRE firefly luciferase reporter (0.5 µg) and the pRL‐TK Renilla luciferase plasmid (0.05 µg). As the internal control, the pRL‐TK Renilla luciferase plasmid was employed. The Dual‐Luciferase Reporter Assay Kit (Promega) was utilized to evaluate the activities of firefly and Renilla luciferase after cells were treated as specified. The firefly to Renilla luciferase ratio was used to derive the relative luciferase activity.

### 4.12 vRNA and plasmid transfection

5ʹPPP‐RNA (vRNA) was generated from A549 cells infected with influenza virus A/PR/8/34 (H1N1) (MOI = 0.1). Infected cells were transfected with 500 ng/mL vRNA using Lipofectamine 2000 (Invitrogen). For plasmid transfection, 100 µL Opti‐MEM medium was used to dilute 500 ng of RIG‐I overexpression plasmid and Lipofectamine 2000. After 20 min of incubation, the mixture was introduced to A549 cells for 6 h. For further incubation, the medium was subsequently replaced with a fresh complete medium.

### 4.13 Statistical analysis

The format of mean ± SD was used to present the results. SPSS 18.0 was utilized to statistically analyze the data. Comparative analyses among more than two groups were conducted using a one‐way analysis of variance followed by Dunnett's test. The significance threshold was established at *p* < 0.05.

## AUTHOR CONTRIBUTIONS

The study was conceptualized and designed by B.‐X. Zhou, J. Li, and X.‐P. Pan. Experimental procedures were carried out by L.‐X. Wang, S.‐S. Yang, and Y.‐Y. Liang. The data analyses were completed by S.‐S. Yang, X.‐Y. Liu, and B.‐X. Zhou. Essential reagents and H1N1 influenza virus strains were obtained by X.‐P. Pan and J. Li. The manuscript was written by B.‐X. Zhou and revised by Y.‐H. Zhang. The final manuscript was a collaborative effort including all authors who also discussed the findings. All authors have read and approved the final manuscript.

## CONFLICT OF INTEREST STATEMENT

All authors declare no conflict of interest.

## ETHICS STATMENT

Animal experiments were conducted in accordance with the Guide for the Care and Use of Laboratory Animals of the National Institutes of Health. All experimental protocols were approved by the Animal Ethics Committee of the Affiliated First Hospital of Guangzhou Medical University (approval number 20230276).

## Supporting information

Supporting information

## Data Availability

The data used to support the findings of this study are available from the corresponding author upon request.
