# Supplementary material for: Pyrogallol protects against influenza A virus‐triggered lethal lung injury by activating the Nrf2–PPAR‐γ–HO‐1 signaling axis
Source: MedComm (2020). 2024 Apr 12;5(4):e531. doi: 10.1002/mco2.531 (PMC11014464; doi:10.1002/mco2.531)
Supplement: Supplementary file 1 — Supporting information [file MCO2-5-e531-s001.doc]

Supplementary Materials for

**Pyrogallol protects against influenza A virus**-**triggered lethal lung injury by activating the Nrf2**-**PPAR**-**γ**-**HO**-**1 signaling axis**

Affiliation

Beixian Zhou^†^, Linxin Wang^†^, Sushan Yang^†^, Yueyun Liang, Yuehan, Zhang, Xuanyu Liu, Xiping Pan^*^, Jing Li^*^

†These authors contributed equally to this work.

*Corresponding author

E-mail: lijing82@gzhmu.edu.cn (J. Li), xppan116@sina.com (X. Pan)


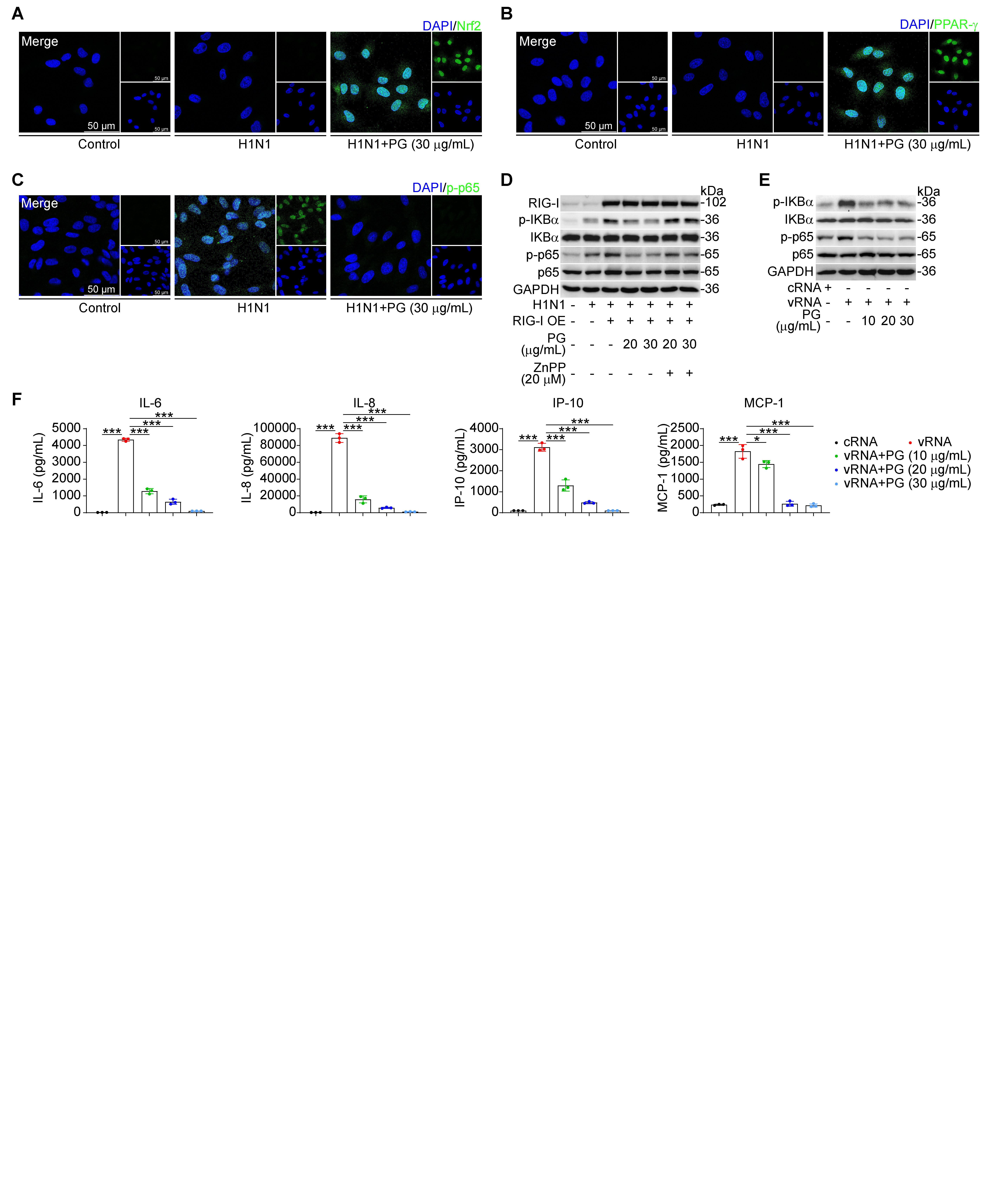


**Figure S1.** Blockade of HO-1 abrogated the suppressive effects of pyrogallol on RIG-I-NF-κB signaling. (A-C) Immunofluorescence detection of the nuclear localization of Nrf2 (A), PPAR-γ (B), p-p65 (C). (D) Immunoblotting of p-IKBα, IKBα, p-p65, and p65 expression in RIG-I overexpression (OE) plasmid-transfected A549 cells. (E) Immunoblot analysis of p-IKBα and p-p65 expression in vRNA-transfected A549 cells. (F) Levels of pro-inflammatory mediators (IL-6, IL-8, IP-10 and MCP-1) in the culture supernatant of vRNA-transfected A549 cells. ^*^*P* < 0.05, ^**^*P* < 0.01, ^***^*P* < 0.001.

Table S1. List of primary antibodies used in this study.

| Antibody | Cat. # | Manufacturer |
| --- | --- | --- |
| cleaved caspase 3 | #9664 | Cell Signaling Technology |
| p-NF-κB p65 (Ser^536^) | #3033 | Cell Signaling Technology |
| NF‑κB p65 | #8242 | Cell Signaling Technology |
| RIG-I | #4200 | Cell Signaling Technology |
| p-JAK1 (Tyr^1034/1035^) | #74129 | Cell Signaling Technology |
| JAK1 | #3344 | Cell Signaling Technology |
| p-STAT1 (Tyr^701^) | #9167 | Cell Signaling Technology |
| STAT1 | #14994 | Cell Signaling Technology |
| p-STAT2 (Tyr^690^) | #88410 | Cell Signaling Technology |
| STAT2 | #72604 | Cell Signaling Technology |
| PARP | GTX100573 | GeneTex |
| active caspase 3 | GTX22302 | GeneTex |
| HO-1 | 10701-1-AP | Proteintech |
| GAPDH | AB2000 | Abways |
| SpC | DF6647 | Affinity Biosciences |
| IL-6 | DF6087 | Affinity Biosciences |
| TNF-α | AF7014 | Affinity Biosciences |
| IL-8 | DF6998 | Affinity Biosciences |
| MCP-1 | DF7577 | Affinity Biosciences |
